# Decoding the impact of night/day shiftwork on well-being among healthcare workers

**DOI:** 10.1038/s41598-024-60882-1

**Published:** 2024-05-04

**Authors:** Lawrence Ejike Ugwu, Erhabor Sunday Idemudia, Maria-Chidi Christiana Onyedibe

**Affiliations:** 1 Faculty of Humanities, North-West University South Africa, Potchefstroom, South Africa; 2 University of Nigeria, Nsukka, Nigeria

**Keywords:** Quality of life, Work schedule, Psychological well-being, Physical health, Environmental conditions, Social relationship, Social support, Psychology, Human behaviour

## Abstract

This study delved into the complex effects of work schedules on the well-being of healthcare professionals, spotlighting Nigeria’s medical landscape. A diverse cohort of 387 participants, spanning doctors, nurses, pharmacists, and laboratory technicians or scientists, formed the research base, with the majority being women (67.7%), with a mean age of 34.67 years. Professionals self-reported their predominant schedules to gauge work patterns, classifying them as day or night shifts. The World Health Organization Quality of Life Brief Version (WHOQOL-BREF) tool assessed the quality of life across the physical, psychological, social relationship, and environmental domains. Psychological distress was measured using the Depression, Anxiety, and Stress Scales (DASS), and perceived social support was evaluated via the Multidimensional Scale of Perceived Social Support (MSPSS). A cross-sectional design was adopted, and the study employed moderated mediation analysis using SmartPLS 4.0. The results underscored the significant ramifications of night shifts on environmental and physical well-being. Psychological health and social relationships were better among day shift than night shift workers. There was a pronounced correlation between night shifts and heightened levels of anxiety, stress, and depression. The mediating role of psychological distress and the moderating influence of social support in these relationships were evident. This study offers invaluable insights into the role of work schedules in shaping the well-being of healthcare professionals, emphasising the protective role of social support and the unique challenges faced by migrant health workers.

## Introduction

Globally, health workers confront a spectrum of professional challenges, with erratic working hours compromising their work-life balance and degrading their quality of life. The impact of work schedules on quality of life, although explored^1,2^, has largely been confined to sectors within developed countries, leaving a stark gap in our understanding of health workers’ experiences in developing contexts. This oversight is critical; in developing nations, including Nigeria, health workers face exacerbated challenges due to limited resources, overwhelming patient loads, and inadequate infrastructure. These conditions, likely intensifying the adverse effects of demanding work schedules on quality of life, underscore the necessity for targeted investigation^3^.

The COVID-19 pandemic has starkly revealed global health systems’ fragilities, particularly in underdeveloped regions where vulnerable populations face heightened risks due to inequitable healthcare access. Studies by Tonkikh et al.^4^ and Rowlands et al.^5^ underscore the dire situations in densely populated areas and health systems of Africa and South Asia, plagued by resource scarcities such as insufficient intensive care units and a lack of healthcare workers. Furthermore, Luo et al.^6^ highlight the critical mental health toll on healthcare professionals, emphasising the necessity for support. The added strain of responding to COVID-19 amidst already overloaded systems and evolving public health policies emphasised the urgent need to focus on health professionals’ well-being and quality of life.

Quality of life, encompassing human well-being and its societal and individual implications, has been widely recognised as a multifaceted concept influenced by various factors^7,8^. The pursuit of improving quality of life underscores its role in advancing health and well-being and guiding policy and healthcare interventions^9^. This study focuses on health professionals in Nigeria^10^, a demographic pivotal to the healthcare system yet facing unique challenges, aiming to uncover strategies that enhance well-being within this crucial sector^10–13^.

The literature reveals a robust link between shift work, especially night shifts, and mental health issues among health workers attributed to disruptions in normal sleep patterns^14,15^. This relationship highlights the need for intervention programs tailored to shift workers to mitigate stress-related health issues^16^. Despite these insights, the specific dynamics of how work schedules, particularly night versus day shifts, impact psychological distress and overall quality of life in health workers in developing contexts like Nigeria require further exploration.

Addressing stress among healthcare shift workers calls for a comprehensive approach, integrating organisational, behavioural, and supportive interventions^17–19,29,30^. Social support emerges as a critical factor, significantly buffering the psychological impact of irregular work schedules^20,21,31,32^. This backdrop frames our study within Lazarus and Folkman’s Stress–Strain Coping Support Model^22^, offering a lens to view the interplay of work schedules, psychological distress, coping strategies, and social support in affecting quality of life.

In Nigeria, the professional conditions for health workers highlight a pressing need for research focused on their well-being^33,34^. The nuanced understanding of work schedules’ impact on quality of life and psychological distress remains sparse, particularly in challenging settings^36,37^.

**Figure 1:**
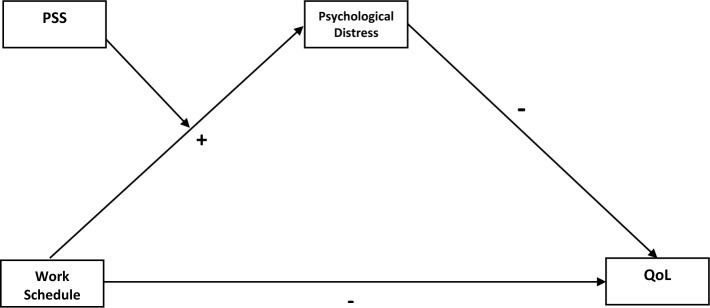
Conceptual framework work schedule, perceived social support, psychological distress and quality of life. *Note*: *PSS* Perceived social support, *QoL* Quality of Life, “ + ”indicate positive relationship; “-”indicates negative relationship.

The conceptual framework (see Fig. 1) elucidated the negative impact of work schedules (night shift) on healthcare workers’ psychological well-being, elevating levels of depression, anxiety, and stress, which in turn detrimentally affects their quality of life. Grounded in empirical evidence, studies like Kang, Noh, and Lee^14^ and Jiang et al.^15^ illustrate the direct relationship between work schedule (shift work) and increased psychological distress. The mediating role of this distress in affecting quality of life is supported by the work of Moons, Budts, and De Geest^7^. Furthermore, perceived social support is identified as a crucial moderating factor that can buffer the adverse effects of work schedules on quality of life, as reflected in the findings of Bambra et al.^17^ and the theoretical underpinnings of Lazarus and Folkman^22^. This support reduces the immediate stress caused by irregular work hours and indirectly enhances overall quality of life, aligning with the WHOQOL Group's^38^ comprehensive assessment approach. This framework emphasises the importance of enhancing social support systems within healthcare settings to mitigate work-induced psychological distress and improve workers' quality of life.

### Objectives of the study

This study aims to elucidate the negative relationship between night and day shift work schedules and the quality of life among Nigerian health workers. It focuses on clarifying the adverse relationship between night and day shift work schedules and their overall well-being. It aims to not only illuminate the direct positive correlation between shift work schedules and psychological distress—encompassing depression, anxiety, and stress—but also to dissect the role of psychological distress as a crucial mediating factor that links work schedules to quality of life, offering a deeper understanding of the mechanisms through which work conditions affect health workers’ well-being. Additionally, the investigation into social support as a moderating variable underlines the essential role of social networks and support systems in buffering the detrimental impacts of shift work on psychological distress and, consequently, on the quality of life. This dual focus on both mediation and moderation underlines the importance of comprehensive support mechanisms in mitigating the negative outcomes associated with occupational stress among health professionals in Nigeria.

## Methods

### Participants

The study was conducted from August 2022 to March 2023 in Southeast Nigeria, covering health workers in General Teaching Hospitals, Specialist Hospitals, and Private hospitals. This timeframe and selection of sites were intended to capture a larger sample size in the region. We employed a purposive sampling strategy, focusing on certified healthcare professionals engaged in patient care. Inclusion criteria were certification in a healthcare profession, proficiency in English, and informed consent to participate. Exclusion criteria targeted non-certified workers, administrative staff, not involved in direct patient care, and those unable or unwilling to consent.

To ensure adequate power for detecting significant effects in moderated mediation regression analysis exploring the association between work schedules, psychological distress, perceived social support and quality of life, we calculated the required sample size using G*Power (Version 3.1.9.7^23^. The power analysis was based on linear multiple regression parameters, aiming for 95% power (1−β = 0.95) and an alpha level 0.05 to identify moderate effect sizes. Anticipated effect sizes were derived from preliminary studies in similar populations^24^. This analysis indicated a required sample size of approximately 129 participants. Our study exceeded this threshold with a total sample of 387 patients, ensuring sufficient power to detect relevant relationships between work schedules, psychological distress, perceived social support and quality of life.

The sample comprised 387 healthcare professionals, including 262 women (67.7%) and 125 men (32.3%). Two-hundred and nine (269, 69.5%) were married, and 118 (30.5%) were single. Hundred and sixteen (30%) worked in the General Hospitals, 196 (50.6%) worked in Private Hospitals, and 75(19.4%) worked in Teaching Hospitals. The participants’ ages ranged from 20 to 66 years, with a mean age of 34.67 (SD = 10.59). The professionals were from diverse disciplines within the healthcare field: 111 (28.7%) were doctors, 140 (36.2%) were nurses, 82 (21.2%) were pharmacists, and 54 (14%) were laboratory technicians or scientists.

### Instruments/material

#### Work schedules

Work Schedules: Participants’ work schedules were categorised as "day shift" or "night shift"^39^. This was determined based on the self-reported predominant work hours. The day shift was defined as working predominantly between 7 AM and 7 PM, whereas the night shift was defined as working predominantly between 7 PM and 7 AM. Participants were asked to select the category that best represented their typical work schedule over the past month. It is important to note that categorising work schedules into day and night shifts is standard in research on shift work and its impacts. However, it does not capture the full range of shift work patterns (e.g. rotating shifts, split shifts). This simplification was necessary for this study, but it may limit the generalizability of the findings to other shift work patterns.

This measure was used as a primary independent variable in the study, and it was hypothesised that work schedules (day shift vs night shift) would be related to several of the dependent variables under investigation. As such, statistical controls were implemented to account for potential confounding factors associated with shift work, such as the number of hours worked per week and job tenure.

#### The WHOQOL-BREF

World Health Organization Quality of Life Brief Version** (**WHOQOL-BREF)^38^ will be used to assess Quality of Life. The WHOQOL-BREF is a 26-item instrument; for this study, we used only the four domains (24-items): physical health (seven items), psychological health (six items), social relationships (three items), and environmental health (eight items). The physical health domain includes activities of daily living, dependence on medical treatment, energy and fatigue, mobility, pain and discomfort, sleep, and work capacity. The psychological health domain includes bodily image and appearance, negative feelings, positive feelings, self-esteem, spirituality, and concentration. The social relationships domain includes personal relationships, social support, and sexual activity. The environmental health domain includes finances, physical safety, access to health services, home environment, opportunities for acquiring new information and skills, leisure activities, physical environment, and transport^25^.

#### Depression, anxiety and stress scales

The DASS is a 21-item Depression Anxiety and Stress Scale developed by Lovibond and Lovibond^26^ was used in this study to measure psychological distress. The DASS is a 21-item self-administered questionnaire designed and derived from the original DASS-42 to measure the magnitude of three negative emotional states: depression, anxiety, and stress.

#### Multidimensional scale of perceived social support

The Multidimensional Scale of Perceived Social Support (MSPSS) was developed by Zimet et al.^27^. It is a 12-item scale that measures perceived support from three domains: family, friends and significant other.

### Design and statistics

The study will use cross-sectional design and moderated mediation regression analysis using SmartPLS 4.0 software, a structural equation modelling tool that uses partial least squares (PLS) regression methods. The analysis was guided by Hayes’ Process Model 7, a framework that allows for examining how another variable may moderate the effect of an independent variable on a dependent variable at different stages in the mediating process.

In our model, the independent variable (IV), the dependent variable (DV), the mediator (M), and the moderating variable (W) were all included. The analysis consisted of several steps:

First, we examined the direct effect of the IV on the DV while controlling for the mediator.

We then calculated the index of moderated mediation, which quantifies the extent to which the indirect effect is conditional upon the moderator. This index provides information about the change in the indirect effect of a one-unit increase in the moderator. We used bootstrapping with 5,000 resamples to generate confidence intervals for the index of moderated mediation.

This analytical strategy allowed us to explore the complex interplay between variables and to understand whether and how the mediation effect varies across different levels of the moderating variable.

### Measurement and structural model

The psychometrics of the tools employed exhibit strong reliability coefficients. From a pilot study conducted by the researchers, the WHOQOL-BREF demonstrated a reliability coefficient of 0.79. The DASS, encompassing various dimensions, yielded reliability values ranging from 0.71 to 0.89. The MSPSS, ensuring its consistency in gauging perceived social support, recorded a reliability coefficient of 0.81.

The structural equation model’s suitability was assessed using various indices outlined^28^. The chi-square test, which evaluates the hypothesis that the model perfectly fits the data, yielded a non-significant result (*χ*2 = 321.362, *p* < 0.061) with 105 degrees of freedom.

Key indicators pointed towards a strong fit: the RMSEA was 0.041, below the accepted threshold of 0.05. The SRMR, at 0.076, was also below its respective benchmark of 0.08.

The GFI (0.91) and the AGFI (0.93) surpassed the standard cut-off of 0.90. Other indicators like the NFI (0.94) and TLI (0.95) exceeded their respective benchmarks of 0.90 and 0.95. The CFI, at 0.952, was just above the 0.95 threshold, indicating an acceptable fit. Lastly, the PGFI was 0.90, showcasing the model’s decent parsimony.

### Ethics approval and consent to participate

The study has been approved by the University of Nigeria Nsukka Internal Review Board (UNN/HREC/202218607AR). The authors affirm that this study was conducted following the ethical standards of the responsible committee on human experimentation (institutional and national) and the Helsinki Declaration of 1975, as revised in 2013. Informed consent was obtained from all the participants.

## Result

The correlation results (see Table 1) show that age is moderately to slightly negatively associated with quality of life in the physical, social, and environmental domains, but this does not extend to the psychological domain. Men tend to have a stronger connection with psychological domains compared to women. Being single is associated with a more pronounced negative impact on all quality-of-life domains. Higher levels of education are linked to a lesser negative impact on the quality of life across the psychological, social relationship, and environmental domains, though this does not apply to the physical domain. Concerning work-related factors, average working hours slightly negatively impact the physical quality of life. Daytime work schedules have a moderate to slight negative correlation with quality of life in psychological, social relationship, and environmental domains. Meanwhile, physical quality of life has a slight yet significant negative correlation with perceived social support and a moderate negative relationship with psychological distress, including depression, anxiety, and stress. The psychological domain of quality of life has a moderate negative correlation with perceived social support and a slight negative correlation with psychological distress, notably depression. Social relationships in quality of life are moderately negatively affected by perceived social support and slightly by psychological distress, such as depression and anxiety. The environmental aspect of quality of life moderately suffers from the negative impacts of perceived social support and is negatively influenced by psychological distress, including depression, anxiety, and stress.

**Table 1:** Descriptive statistics and correlation matrix.

				1	2	3	4	5	6	7	8	9	10	11	12	13	14
1	Age	34.67	10.59	–													
2	Sex			− 0.13**	–												
3	Marital status			0.44**	0.21**	–											
4	Highest educational level			0.36**	− 0.27**	0.17**	–										
5	Average working hour	6.86	4.03	0.29**	− 0.14**	0.16**	0.19**	–									
6	Work schedule			− 0.21**	0.04*	− 0.15**	− 0.18**	− 0.10**	–								
7	Perceived Social Support	66.51	12.35	0.08**	0.01	0.13**	0.02	0.05**	0.01	–							
8	Depression	4.92	3.86	− 0.01	0.13**	− 0.10**	0.15**	− 0.09**	0.03	− 0.10**	–						
9	Anxiety	4.53	3.63	0.05**	0.01	0.12**	-0.15**	-0.08**	0.17**	0.05**	0.67**	–					
10	Stress	6.03	3.66	− 0.01	0.07**	0.18**	− 0.06**	− 0.05**	0.04*	− 0.00	0.78**	0.62**	–				
11	Physical domain	20.47	2.85	− 0.11**	− 0.07	− 0.04*	0.01	− 0.04*	0.04	− 0.11**	− 0.17**	− 0.29**	− 0.08**	–			
12	Psychological domain	21.20	3.62	− 0.01	− 0.25**	− 0.04*	− 0.09**	− 0.00	− 0.15**	− 0.20**	− 0.14**	− 0.03	− 0.01	0.33**	–		
13	Social Relationship domain	15.06	1.77	− 0.14**	0.03	− 0.07**	− 0.08**	− 0.03	− 0.21**	− 0.34**	− 0.05**	− 0.06**	− 0.03	0.31**	0.18**	–	
14	Environmental domain	16.85	2.73	− 0.22**	− 0.01	− 0.22**	− 0.05**	− 0.04	− 0.13**	− 0.31**	− 0.16**	− 0.09**	− 0.22**	0.28**	0.29**	0.42**	–

* = p < 0.05, ** = p < 0.001; Sex –(dummy coded ‘0’ male, ‘1’-female); marital status (dummy coded ‘0’ single, ‘1’-married); highest educational level (1-Diploma, 2-Honours, 3- Postgraduate degree); work schedule (dummy coded ‘0’- day shift, ‘1’- night shift).

Our exploration of the connection between work schedules (day shift and night shift) and quality of life (environmental, physical, psychological and social relationship) (see Table 2) showed that work schedules (night shift) were significantly related to environmental well-being (β = 0.71) and physical health (β = 0.54). Work schedule (day shift) was significantly related to psychological health (β =  − 1.20) and social relationships (β = 0.73); this thus supports the first hypothesis.

**Table 2:** Direct relationships.

Direct relationships	Coefficient	T-values	p-value
Work schedule—> environmental	0.71	6.84	0.001
Work schedule—> physical health	0.54	5.13	0.001
Work schedule—> psychological health	− 1.20	7.01	0.001
Work schedule—> social relationships	0.73	10.23	0.001
Work schedule—> anxiety	− 3.78	5.18	0.001
Work schedule—> depression	− 1.46	1.85	0.032
Work schedule—> stress	− 3.90	5.45	0.001
Anxiety—> environmental	0.03	1.97	0.024
Anxiety—> physical health	− 0.30	17.08	0.001
Anxiety—> psychological health	0.12	3.94	0.001
Anxiety—> social relationships	0.00	0.22	0.412
Depression—> Environmental	0.01	0.70	0.241
Depression—> physical health	− 0.07	3.12	0.010
Depression—> psychological health	− 0.38	8.67	0.001
Depression—> social relationships	0.03	1.94	0.026
Stress—> environmental	-0.20	10.07	0.001
Stress—> physical health	0.18	8.75	0.001
Stress—> psychological health	0.23	6.78	0.001
Stress—> social relationships	-0.02	1.23	0.110
Perceived social support x work schedule—> anxiety	-0.04	3.52	0.001
Perceived social support x work schedule—> depression	-0.02	1.59	0.056
Perceived social support x work schedule—> stress	-0.06	5.11	0.001

Work schedule (dummy coded ‘0’- day shift, ‘1’- night shift).

The connection between work schedules and psychological distress (depression, anxiety, and stress) showed that work schedules (night shift) significantly influence these outcomes. Specifically, night shift workers are associated with an increase in anxiety (β = 3.78), stress (β = 3.9) and depression (β = 1.46); this supports the second hypothesis.

Depression’s effects, while not significant for environmental well-being, manifested as negative for both physical (β =  − 0.07) and psychological health (β =  − 0.38). A mild positive association was observed between depression and social relationships (β = 0.03).

Upon deeper investigation into the consequences of anxiety on various quality-of-life domains, it emerged that heightened anxiety marginally augments environmental well-being (β = 0.03). Notably, anxiety has a pronounced negative impact on physical health (β =  − 0.30). In the domain of psychological health, a rise in anxiety is linked to worsened outcomes, evidenced by a coefficient of β = 0.12, and significant at the p < 0.01 level. Regarding social relationships, the influence of anxiety (β = 0.00) remains statistically non-significant.

Stress demonstrated a negative effect on environmental well-being (β =  − 0.20) while positively influencing both physical (β = 0.18, p < 0.01) and psychological health (β = 0.23). The relationship between stress and social relationships did not achieve statistical significance. This complex result has made the findings unclear, not less likely for generalisation.

When considering the moderating effect of perceived social support on the relationship between work schedules and psychological distress dimensions, it was found that this interaction significantly diminishes anxiety (β =  − 0.04) and stress (β =  − 0.06). However, its moderating effect on depression was not statistically significant, thus also creating an inconclusive finding on the forth hypothesis.

In Table 3, we have delved deep into understanding the influence of the interplay between perceived social support and work schedules on various health dimensions, particularly considering the mediating roles of stress, depression, and anxiety.

**Table 3:** Specific indirect effect.

Specific indirect effect	Coeff	SE	LLCI	ULCI	P values
Perceived social support x work schedule—> Anxiety—> environmental	− 0.001	0.001	-0.002	0.000	0.035
Perceived social support x work schedule—> Anxiety—> physical	0.011	0.003	0.006	0.017	0.001
Perceived social support X work schedule—> Anxiety—> psychological	− 0.005	0.002	− 0.008	− 0.002	0.006
Perceived social support x work schedule—> Anxiety—> social relationships	0.000	0.000	-0.001	0.001	0.415
Perceived social support x work schedule—> depression—> environmental	0.000	0.000	-0.001	0.000	0.286
Perceived social support x work schedule—> depression—> physical	0.001	0.001	0.000	0.003	0.074
Perceived social support x work schedule—> depression—> psychological	0.007	0.004	0.000	0.014	0.060
Perceived social support x work schedule—> depression—> social relationships	− 0.001	0.001	− 0.002	0.000	0.149
Perceived social support x work schedule—> stress- > environmental	0.011	0.002	0.007	0.000	0.001
Perceived social support x work schedule—> stress—> physical	− 0.01	0.002	− 0.014	− 0.006	0.001
Perceived social support x work schedule—> stress—> psychological	− 0.013	0.003	− 0.018	− 0.008	0.001
Perceived social support x work schedule—> stress—> social relationships	0.001	0.001	0.000	0.002	0.119

Perceived social support, *SE *Standard Error, *LLCI* Lower Limit Confidence Interval, *ULCI *Upper Limit Confidence Interval.

Our findings suggest that stress significantly mediates, especially when considering the impact on environmental well-being. When employees experience variations in their work schedules and simultaneously perceive varying levels of social support, this changes stress levels, significantly affecting their environmental well-being (β = 0.011, p = 0.001).

However, when we turn our attention to depression as a potential mediator for the same relationship, the effect is not statistically significant. The coefficient is β = 0.000, with a significance level of p = 0.286, indicating that the path through depression does not substantially influence environmental well-being.

Anxiety, another pivotal mental health dimension, also showcases its mediating ability. Our analysis revealed a significant negative indirect effect of the perceived social support and work schedule interaction on psychological health via anxiety (β =  − 0.005, p = 0.006). Similarly, stress emerges as a significant mediator, negatively impacting psychological health (β =  − 0.013, p = 0.001). However, its influence on social relationships is not statistically significant (β = 0.001, p = 0.119).

Further, the mediation effect of depression on physical health is observed to be on the cusp of significance (β = 0.001, p = 0.074). Anxiety’s role as a mediator between the interaction and environmental well-being is evident and significant (β =  − 0.001, p = 0.035), whereas its effect on social relationships does not achieve statistical significance.

One of the most pronounced findings is the strong negative indirect effect of the perceived social support and work schedule interaction on physical health, mediated by stress (β =  − 0.01, p = 0.001). Additionally, while depression’s mediating effect on psychological health is positive, it is marginally significant (β = 0.007, p = 0.060). When mediating the relationship with physical health, anxiety’s influence emerged as significant and positive (β = 0.011, p = 0.001).

This investigation explains how perceived social support and work schedules, two pivotal factors in today’s work environment, interact and influence various health dimensions. The mediating roles of stress, depression, and anxiety bring forth an intricate tapestry of relationships, emphasising the importance of considering multiple pathways when exploring workplace well-being.

The complex interplay between work schedules (day and night shift), psychological distress (anxiety, depression, and stress), and perceived social support levels sheds light on how different domains of quality of life (environmental, physical, psychological, and social relationships) are influenced (see Table 4).

**Table 4:** Probing Moderated Indirect Relationships (Slope Analysis).

Probing moderated indirect relationships	Indirect effect	LLCI	ULCI	P values
Work Schedule—> anxiety—> environmental (higher level of PSS)	0.02	0.003	0.047	0.056
Work schedule—> anxiety—> environmental (lower level of PSS)	0.05	0.008	0.092	0.025
Work schedule—> anxiety—> physical (higher level of PSS)	− 0.22	− 0.317	− 0.123	0.000
Work schedule—> anxiety—> physical (lower level of PSS)	− 0.50	− 0.606	− 0.403	0.000
Work schedule—> anxiety—> psychological (higher level of PSS)	0.09	0.044	0.151	0.003
Work schedule—> anxiety—> psychological (lower level of PSS)	0.21	0.121	0.311	0.001
Work schedule—> anxiety—> social relationships (higher level of PSS)	0.00	− 0.009	0.012	0.414
Work schedule—> Anxiety—> social relationships (lower level of PSS)	0.00	-0.021	0.028	0.412
Work—> schedule—> depression Environmental (higher level of PSS)	0.00	− 0.006	0.007	0.489
Work schedule—> depression—> environmental (lower level of PSS)	0.01	− 0.008	0.02	0.263
Work schedule—> depression—> physical (higher level of PSS)	− 0.00	− 0.028	0.024	0.482
Work schedule—> depression—> physical (lower level of PSS)	− 0.03	− 0.066	− 0.007	0.031
Work schedule—> depression—> psychological (higher level of PSS)	− 0.00	− 0.129	0.12	0.481
Work schedule—> depression—> psychological (lower level of PSS)	− 0.18	− 0.311	− 0.046	0.015
Work schedule—> depression—> social relationships (higher level of PSS)	0.00	− 0.013	0.012	0.483
Work schedule—> depression—> social relationships (lower level of PSS)	0.02	0.001	0.039	0.104
Work schedule—> stress—> environmental (higher level of PSS)	0.08	0.016	0.148	0.025
Work schedule—> stress—> environmental (lower level of PSS)	− 0.19	− 0.26	− 0.118	0.001
Work schedule—> stress—> physical (higher level of PSS)	− 0.07	− 0.138	− 0.013	0.027
Work schedule—> stress—> physical (lower level of PSS)	0.17	0.108	0.237	0.000
Work schedule—> stress—> psychological (higher level of PSS)	− 0.09	− 0.181	− 0.018	0.031
Work schedule—> stress—> psychological (lower level of PSS)	0.22	0.135	0.31	0.001
Work schedule—> stress—> social relationships (higher level of PSS)	0.01	− 0.002	0.02	0.158
Work schedule—> stress—> social relationships (lower level of PSS)	− 0.02	− 0.044	0.005	0.122

Perceived social support, *SE* Standard Error, *LLCI* Lower Limit Confidence Interval, *ULCI* Upper Limit Confidence Interval.

When considering anxiety as the intermediary, those with heightened anxiety levels show a subtle but discernible link between their work schedules and environmental (quality of life), represented by an effect of β = 0.02 (95% CI [0.003, 0.047]). This connection intensifies slightly for individuals with lower anxiety levels, where the effect is β = 0.05 (95% CI [0.008, 0.092]).

**Figure 2:**
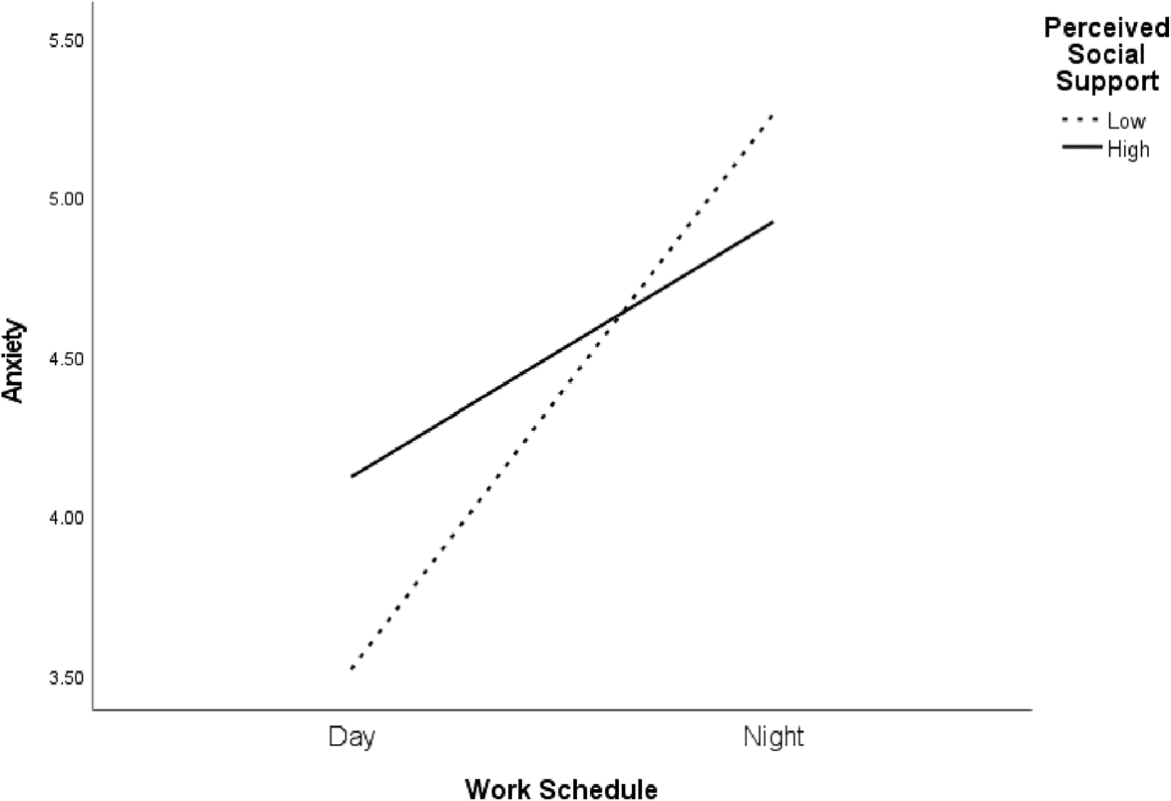
Interaction graph between work schedules, perceived social support, and anxiety.

PSS levels reveal a negative association between work schedules and physical health. For those with higher PSS levels, the effect is β =  − 0.22 (95% CI [− 0.317, − 0.123]), and it becomes even more pronounced for those at the lower end of the PSS spectrum, with an effect of β =  − 0.50 (95% CI [− 0.606, − 0.403]). Additionally, the relationship between work schedules and psychological outcomes, mediated by anxiety, is evident with a value of β = 0.09 (95% CI [0.044, 0.151]) for the higher PSS group. This effect amplifies for those with lower PSS, which stands at β = 0.21 (95% CI [0.121, 0.311]) (see Fig. 2).

**Figure 3:**
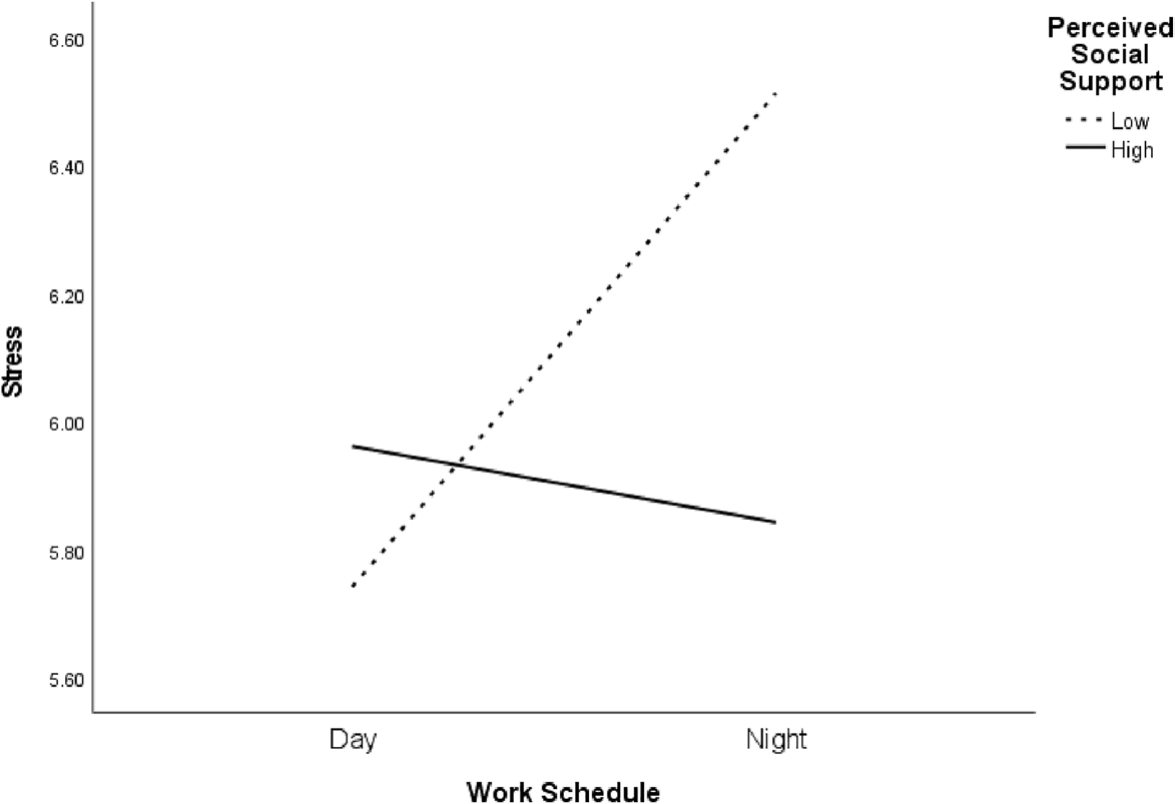
Interaction graph between work schedules, perceived social support, and stress.

Moving on to stress as the mediating factor, higher PSS individuals exhibit a more robust connection between their work schedule and environmental outcomes, marked by β = 0.08 (95% CI [0.016, 0.148]). In contrast, those with lower PSS levels reveal a negative association, denoted by β =  − 0.19 (95% CI [− 0.26, − 0.118]). When considering stress, physical health outcomes show a negative relationship for higher PSS individuals, with an effect of β =  − 0.07 (95% CI [− 0.138, − 0.013]). This trend reverses for the lower PSS group, where the effect is positively marked at β = 0.17 (95% CI [0.108, 0.237]). The psychological domain, mediated by stress, reflects a decreasing influence of work schedule with increased perceived social support for the higher PSS group β =  − 0.09, 95% CI [− 0.181, − 0.018]). For those with lower PSS, the effect is positively marked at β = 0.22 (95% CI [0.135, 0.31]) (See Fig. 3).

In summary, the nature of one’s work schedule and anxiety or stress levels can significantly shape various life outcomes. The direction and strength of these relationships are further shaped by the individual’s perceived stress levels, underscoring the multifaceted nature of human experiences and well-being.

Some noteworthy patterns emerge in exploring how perceived social support influences the relationship between work schedules and various outcomes (see Table 5). When anxiety acts as the mediating factor, an increase in perceived social support slightly diminishes the indirect effect of work schedule on environmental outcomes, as indicated by a value of β =  − 0.001 (95% CI [− 0.002, 0.000], p = 0.035). In contrast, for physical outcomes, the indirect influence of work schedule through anxiety becomes more pronounced with a value of β = 0.011 (95% CI [0.006, 0.017], p < 0.001) as perceived social support rises. The scenario flips for psychological outcomes, where the indirect effect dampens with increased perceived social support, marked by β =  − 0.005 (95% CI [− 0.008, − 0.002], p = 0.006). For social relationships, perceived social support does not seem to play a significant moderating role, as the relationship was not statistically significant.

**Table 5:** Index of moderated mediation.

Index of moderated mediation	Indirect effect	LLCI	ULCI	P values
Perceived social support x work schedule—> anxiety—> environmental	− 0.001	− 0.002	0.000	0.035
Perceived social support x work schedule—> anxiety—> physical	0.011	0.006	0.017	0.000
Perceived social support x work schedule—> anxiety—> psychological	− 0.005	− 0.008	− 0.002	0.006
Perceived social support x work schedule—> anxiety—> social Relationships	0.000	− 0.001	0.001	0.415
Perceived social support x work schedule—> depression—> environmental	0.000	− 0.001	0.000	0.286
Perceived social support x work schedule—> depression—> physical	0.001	0.000	0.003	0.074
Perceived social support x work schedule—> depression—> psychological	0.007	0.000	0.014	0.060
Perceived social support x work schedule—> depression—> social relationships	− 0.001	− 0.002	0.000	0.149
Perceived social support x work schedule—> stress—> environmental	0.011	0.007	0.015	0.000
Perceived social support x work schedule—> stress—> physical	− 0.01	− 0.014	− 0.006	0.000
Perceived social support x work schedule—> stress—> psychological	− 0.013	− 0.018	− 0.008	0.000
Perceived social support x work schedule—> stress—> social relationships	0.001	0.000	0.002	0.119

Considering the mediating effect of depression, the data does not show a significant moderating effect of perceived social support on the indirect relationship between work schedule and environmental outcomes. However, there is a positive but non-significant relationship for physical outcomes, with a value of β = 0.001 (95% CI [0.000, 0.003], p = 0.074). The psychological domain reveals a non-significant relationship with increased perceived social support, as indicated by β = 0.007 (95% CI [0.000, 0.014], (p = 0.060]). Social relationship outcomes, on the other hand, do not show a significant influence.

Lastly, when considering stress as the mediator, the environmental outcomes show a strengthening of the indirect effect with increasing perceived social support, represented by β = 0.011 (95% CI [0.007, 0.015], p < 0.001). Physical outcomes present a decrease in the indirect effect with a value of β =  − 0.01 (95% CI [− 0.014, − 0.006], p < 0.001). Psychological outcomes echo this decreasing trend, more strongly marked by β =  − 0.013 (95% CI [− 0.018, − 0.008], (p < 0.001]). Though slightly positive for social relationships, the effect remains statistically insignificant.

The impact of work schedules on various life outcomes channelled through anxiety, depression, and stress is delicately shaped by the levels of perceived social support an individual receives. The effects, whether amplifying or dampening, underscore the intricate interplay between work conditions, emotional well-being, and the cushioning effect of social support; thus, this supports H4a & b.

## Discussion

The nexus between work schedules, psychological distress, and quality of life of health professionals is particularly pertinent when exploring the experience of health workers in Nigeria. In developing nations like Nigeria, health systems often operate under resource constraints, characterised by high patient loads, inadequate infrastructure, and frequent staff shortages stemming from the high migration rate of healthcare workers.

Our finding that work schedules (night shift) influenced quality of life is consistent with previous studies^29,30^ as hypothesised. This could further exacerbate the observed associations between night shifts and deteriorated environmental well-being and physical health. In addition, the average working hours were almost 7–10 h. This shows its negative impact on the physical health of workers. On the other hand, day shift workers in developing contexts might still benefit from more stable social interactions and better psychological health due to more *normalised* working hours.

The heightened susceptibility of work schedules, especially for night shift workers in Nigeria, to psychological distress, specifically anxiety, stress, and depression, further magnifies the challenges experienced in their working environment. The healthcare environment, already strained by factors like infectious disease outbreaks (for example, the COVID-19 pandemic), inadequate training, and often insufficient protective measures, can make night shifts even more taxing. The results corroborate our second hypothesis and the narrative of studies^31^ and^32^, highlighting the immense pressure on health systems, especially during crises like the COVID-19 pandemic.

The mediating role of psychological distress in the relationship between work schedules and quality of life, as hypothesised, is supported. This is of paramount importance in the context of developing countries. As observed in our study, the mixed effects of stress and anxiety on quality-of-life domains emphasise the complex interplay of workplace demands^33^ and individual coping mechanisms^34^. In nations like Nigeria, where societal and familial structures are robust, the positive impact of stress on social relationships might be more pronounced as individuals lean on these structures for support.

### The implications of this study

This research delves into the profound challenges healthcare professionals face, particularly emphasising the developing landscape of nations like Nigeria. The study adds to the existing literature on work schedules and the strain night shifts exert on healthcare workers. The need to provide interventions to mitigate the psychological impact on their quality of life and, consequently healthcare institutions. The pressing need for targeted mental health interventions is highlighted, with a recommendation for incorporating regular mental health assessments and stress management techniques^17–19,35,36^.

Furthermore, the study highlights the potential pitfalls migrating health workers face, many of whom grapple with the need for familiar support systems as they navigate new work cultures. The onus, therefore, falls on the health institutions to ensure the implementation of interventions.

In the academic realm, the study identifies gaps in the current understanding of individual coping mechanisms and the complex impacts of different work schedules in sub-Saharan Africa. There is a clear indication that future studies should explore these areas in greater depth. The potential enhancement of medical training curriculums also emerges as a recommendation, advocating for including modules on staff-self-mental well-being care. In essence, by prioritising the well-being of healthcare professionals, this study posits a roadmap towards improved patient care and a more robust healthcare system^37^.

In essence, a discussion on public policies should focus on creating a systemic change that addresses the root causes of mental health issues among healthcare workers. This includes considering shift lengths, rest periods, access to mental health resources, and creating a supportive work environment that fosters resilience and well-being. Integrating these considerations into healthcare delivery aims to cultivate a more humane and effective healthcare system that prioritises the health of both caregivers and recipients.

### Recommendation for future studies

This study sheds light on the challenges healthcare professionals encounter, particularly in regions like Nigeria and for migrants. Night shifts strain their well-being, so institutions are urged to rethink shift structures. Enhanced mental health measures, including regular check-ups, are vital. For migrants, tailored orientation and mentorship are essential in navigating unfamiliar work cultures.

The findings emphasise the importance of social support. Institutions can amplify this by fostering family and community ties. These insights should drive governments to craft policies prioritising healthcare workers’ well-being. Further research is needed on coping mechanisms and diverse work patterns. Embracing cultural sensitivity for migrants and possibly incorporating self-care in training are pressing considerations. Ultimately, prioritising healthcare professionals’ well-being enhances patient care and strengthens the broader healthcare system.

### Limitations

While offering insightful findings, this study possesses limitations that deserve acknowledgement. One primary concern is the potential need for more diversity within the sample. If the study focused mainly on Nigerian participants, including Nigerians who migrated to other countries, this could enrich the findings. The cross-sectional study design captures only a snapshot, possibly overlooking evolving patterns or long-term impacts. Additionally, the reliance on self-reported data can introduce subjective biases. Participants might, consciously or unconsciously, shape their responses based on societal perceptions or memory lapses.

Furthermore, establishing and adopting randomised control trials could improve the cause-and-effect relationships from the data. Finally, cultural norms and values intrinsic to Nigerian society, and shaping perceptions of work, well-being, and social ties, might have to be deeply integrated into the study. Recognising these limitations provides context and offers avenues for refinement in subsequent research.

To address these challenges, we implemented rigorous statistical validation methods to enhance the reliability of our data. We also engaged in thoughtful discussions about the potential influences of cultural norms, acknowledging the limitations of our analysis in capturing the full breadth of cultural complexity.

Our study acknowledges the potential limitation that work schedules, particularly night and irregular shifts, may introduce a significant bias by disrupting the natural sleep–wake cycle of participants. This aspect was not extensively examined, which could affect our findings’ applicability and depth regarding the impact of shift work on healthcare professionals’ mental and physical health. Future research should more thoroughly investigate the role of work schedules in influencing health outcomes to mitigate this limitation.

Also, there is the potential for self-reporting to skew results by amplifying or introducing biases, which underscores the need for a more objective assessment, such as evaluations by healthcare professionals or analysis of medical records, to complement self-reported data. These considerations highlight areas for further research to develop a more comprehensive understanding of the interplay between work schedules, mental health, and overall well-being among healthcare workers.

However, it does not capture the full range of shift work patterns (e.g., rotating shifts, split shifts). This simplification was necessary for this study, but it may limit the generalizability of the findings to other shift work patterns.

Our study’s strengths include its methodological rigour and consideration of a deep cultural context. By shedding light on immediate issues and identifying areas for future research, our work provides valuable insights into the lives of Nigerian healthcare workers.

## Conclusion

This study provides a comprehensive examination of the impact of work schedules on the well-being of healthcare professionals in Nigeria, particularly emphasising the challenges faced by migrants. Night shifts have discernible repercussions on physical and environmental well-being in healthcare settings. Moreover, the pivotal role of social support as a buffer against work-related stressors emerged prominently. While the findings underscore the pressing need for institutional and policy-level interventions, they also highlight areas for future research. As healthcare systems globally grapple with evolving challenges, prioritising the well-being of their frontline professionals is paramount. This study is a step forward, emphasising the intricate interplay between work conditions, psychological health, and social support in shaping the quality of life for healthcare workers.

One of the standout findings of this research is the critical role social support plays in mitigating job-related challenges. The study suggests a proactive approach by institutions to introduce tailored interventions for their staff. On a broader canvas, this research sends a strong message to governments, especially in developing nations, to craft policies with a laser focus on the well-being of their healthcare workforce.

## Data Availability

The datasets generated and analysed during the current study are not publicly available due to agreement with the participants and are only available from the corresponding author upon reasonable request.
